# Ideal Cereals With Lower Arsenic and Cadmium by Accurately Enhancing Vacuolar Sequestration Capacity

**DOI:** 10.3389/fgene.2019.00322

**Published:** 2019-04-09

**Authors:** Fenglin Deng, Min Yu, Enrico Martinoia, Won-Yong Song

**Affiliations:** ^1^Department of Horticulture, Foshan University, Foshan, China; ^2^Department of Integrative Biosciences and Biotechnology, Pohang University of Science and Technology, Pohang, South Korea; ^3^Institute of Plant Biology, University of Zurich, Zurich, Switzerland

**Keywords:** arsenic, cadmium, food safety, vacuolar sequestration, crop engineering, cell specificity

## Abstract

Cereals are a staple food for many people around the world; however, they are also a major dietary source of toxic metal(loid)s. Many agricultural regions throughout the world are contaminated with toxic metal(loid)s, which can accumulate to high levels in the grains of cereals cultivated in these regions, posing serious health risks to consumers. Arsenic (As) and cadmium (Cd) are efficiently accumulated in cereals through metal transport pathways. Therefore, there is an urgent need to develop crops that contain greatly reduced levels of toxic metal(loid)s. Vacuolar sequestration of toxic metal(loid)s is a primary strategy for reducing toxic metal(loid)s in grains. However, until recently, detailed strategies and mechanisms for reducing toxic metal(loid)s in grain were limited by the lack of experimental data. New strategies to reduce As and Cd in grain by enhancing vacuolar sequestration in specific tissues are critical to develop crops that lower the daily intake of As and Cd, potentially improving human health. This review provides insights and strategies for developing crops with strongly reduced amounts of toxic metal(loid)s without jeopardizing agronomic traits.

## Introduction

Toxic metals and metalloids are widespread in the Earth’s crust. Among them, inorganic arsenic (As) and cadmium (Cd) are regarded as the group-1 carcinogens due to their toxicity, prevalence, and potential for human exposure (Smith et al., 2002; Sarwar et al., 2010; WHO, 2011). Cereal staples such as wheat (*Triticum aestivum*), rice (*Oryza sativa*), barley (*Hordeum vulgare*), and maize (*Zea mays*) are the primary sources of toxic metal(loid)s ingestion.

Rice, which is consumed by people of many nations around the world, is a major source of As and Cd exposure. A large portion of the arable land in the major rice producing and consuming areas of South and Southeast Asia and China is highly contaminated with As and/or Cd (Mandal and Suzuki, 2002; Zhu et al., 2008; Zhao et al., 2010, 2015). Based on the amount of rice consumed and the content of inorganic As in the polished grains, it has been estimated that the populations of Bangladesh and China have a much higher cancer risk from As poisoning than do Japanese, Italian, and American populations (Meharg et al., 2009). Furthermore, wheat- and rice-based infant food represents an important source of As exposure for infants and toddlers (European Food Safety Authority [EFSA],  2014). In addition, rice accounts for approximately 40% of the food-derived Cd exposure in the Japanese population (Watanabe et al., 2000). Cereals and grains are estimated to contribute about 32% of the total Cd intake by the Chinese population based on the national average estimate of exposure (WHO,  2011). In addition, the primary sources of dietary Cd exposure for adults in Europe are cereals and grains (Olsson et al., 2002; WHO, 2011). Thus, cereals represent the most significant dietary source of As and Cd.

Toxic metal(loid)s accumulation in cereals is regulated by both environmental factors and genetic mechanisms in plants. Recent advances in understanding these molecular mechanisms have led to promising avenues for developing ideal cereals with reduced grain As and Cd.

## Movement of as and Cd From the Soil to the Grain

The absorption, root-to-shoot translocation, and (re)distribution of As and Cd are three crucial steps controlling their movement from the soil to the edible organs. During the last decade, considerable advances in As and Cd accumulation mechanisms in plants have been achieved (see the reviews by Mendoza-Cozatl et al., 2011; Clemens et al., 2013; Uraguchi and Fujiwara, 2013; Clemens and Ma, 2016; Li et al., 2016; Awasthi et al., 2017; Chen et al., 2017; Lindsay and Maathuis, 2017). Herein, the mechanisms of As and Cd transport from soil to the grains of cereals, especially in rice were briefly described.

**FIGURE 1:**
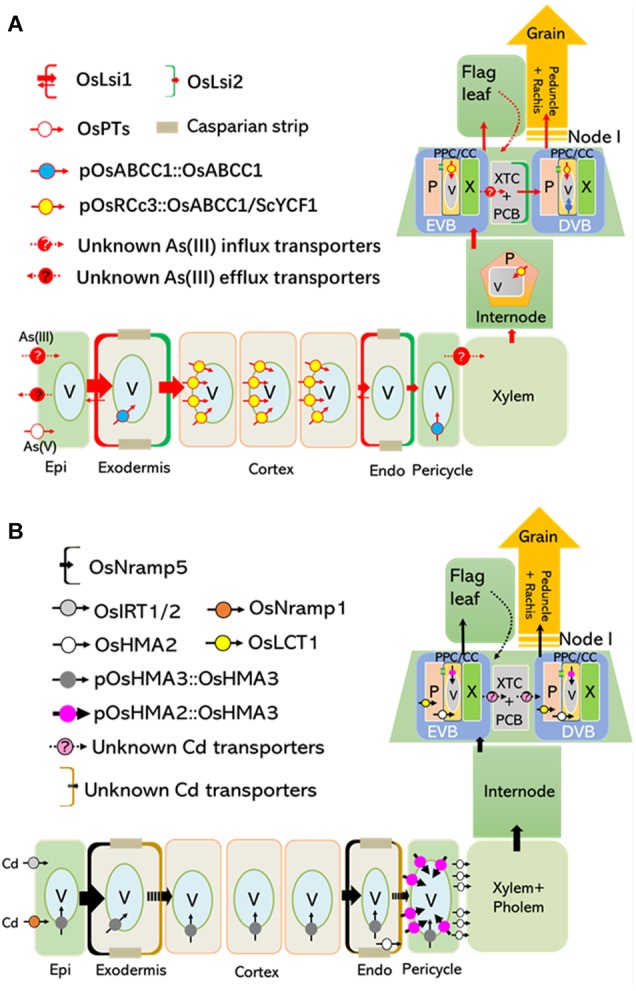
Tissue-specific expression of OsABCC1 and OsHMA3 reduces As and Cd concentrations in the rice grain. Transporters localized at the plasma membrane and tonoplast are critical for root-to-shoot translocation and grain accumulation of As and Cd. **(A)** Arsenic taken up by transporters, such as OsPTs and unknown influx transporters (possibly aquaporins) located at the plasma membrane of the root epidermis, is translocated to shoots and grains, or extruded into the rhizosphere. OsABCC1, a major vacuolar PCs-As transporter, delivers As into vacuoles in phloem companion cells of node I, and inhibits As translocation into rice grains (Song et al., 2014a). Rice plants containing low concentrations of As in their grain were generated by expressing *OsABCC1* and *ScYCF1* specifically in the cortex, internode, and nodes using the *OsRCc3* promoter (Deng et al., 2018). **(B)** Cd is mainly taken up by OsNramp5, which is localized at the distal side of both root exodermis and endodermis cells. In addition, OsIRT1, 2 and OsNramp1 also contribute to Cd uptake. OsHMA3 located at the tonoplast of roots are responsible for the accumulation of Cd within vacuoles and inhibit radial translocation of Cd into the stele. Low Cd-accumulating rice was generated by expressing a functional OsHMA3 transporter under the control of pOsHMA2, a rice root pericycle and nodal phloem-specific promoter (Shao et al., 2018). Epi, epidermis; Endo, endodermis; EVB, enlarged vascular bundles; DVB, diffuse vascular bundles; XTC, xylem transfer cells; PCB, parenchyma cell bridge; NVA, nodal vascular anastomoses; PPC/CC, phloem parenchyma cells and companion cells; P, phloem; V, vacuole.

The uptake and root-to-shoot translocation of arsenite [As(III)], the predominant form in anaerobic soil where rice grow, is largely depends on two silicon (Si) transporters, OsLsi1 and OsLsi2 (Ma et al., 2008). The distally localized OsLsi1 in root exodermal and endodermal cells is responsible for the influx of As(III) into these two cell layers, while proximally localized OsLsi2 at the same cells is required for As(III) efflux toward the stele (Figure 1A; Ma et al., 2008). *OsLsi2* is also highly expressed in the node, a most important organ for As storage and distribution into the rice grain (Chen et al., 2015). Particularly, OsLsi2 is located in parenchyma cells bridging the border between the enlarged vascular bundles (EVBs) and diffuse vascular bundles (DVBs), and involved in the intervascular transfer of As(III) (Figure 1A; Chen et al., 2015; Yamaji et al., 2015). Knockout of *OsLsi1* significantly reduces As(III) uptake, and disruption of *OsLsi2* reduces As translocation to the shoot, as well as the redistribution to the grains (Figure 1A; Ma et al., 2008; Chen et al., 2015). Recently, a transcriptional factor, OsARM1 (Arsenite Responsive MYB 1), was identified as a negative regulator for As(III) transport in rice through directly suppressing the expression of *OsLsi1*, *OsLsi2*, and *OsLsi6*, an aquaporin with As(III) permeability (Ma et al., 2008; Wang et al., 2017). On the other hand, Arsenate [As(V)] is the other form of inorganic As which can be facilitated into root cells by phosphate transporters and subsequently deoxygenated to As(III) by As(V) reductases (Chao et al., 2014; Shi et al., 2016; Cao et al., 2017; Chen et al., 2017; Xu et al., 2017.

In rice, Cd is mainly taken up by OsNramp5, a polar-localized plasma membrane protein belonging to the Natural Resistance-Associated Macrophage Protein family required for manganese (Mn) uptake (Figure 1B; Ishikawa et al., 2012; Sasaki et al., 2012). The T-DNA insertion mutants of *OsNramp5* showed largely reduced yield (Sasaki et al., 2012), while *OsNramp5* knockout lines developed by Ion-beam irradiation or CRISPR/Cas9 system did not show any reduced grain yield (Ishikawa et al., 2012; Tang et al., 2017). Additional investigations are required to clarify this discrepancy. OsNramp1, OsIRT1, and OsIRT2 are also implicated in Cd uptake under specific conditions, but their contribution seems weak compared to that of OsNramp5 (Figure 1B; Nakanishi et al., 2006; Takahashi et al., 2011). After its uptake, Cd is partially translocated from roots to shoots through phloem loading mediated by OsHMA2, a root pericycle-localized heavy metal-transporting P-type ATPase required for translocating Zn to the shoot (Figure 1B; Satoh-Nagasawa et al., 2012; Takahashi et al., 2012; Yamaji et al., 2013). At the reproductive growth stage, OsHMA2 is highly expressed in the phloem at nodal EVBs and DVBs, demonstrating its critical role in loading Cd into seeds through the nodal vasculature (Yamaji et al., 2013). Furthermore, the plasma membrane-localized low-affinity cation transporter 1 (OsLCT1) exports Cd from the nodal phloem parenchyma cells into the sieve tube, ultimately contributing to Cd deposition into grains (Figure 1B; Uraguchi et al., 2011).

## Vacuolar Sequestration Limits as and Cd Allocation Into Grain

**FIGURE 2:**
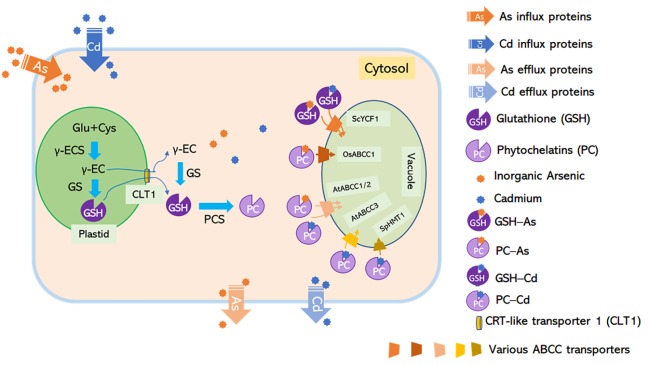
C-type ATP-binding cassette transporters mediate non-protein thiol-dependent As and Cd vacuolar sequestration. Glutathione synthesized by GSH1 is translocated from plastids to the cytosol through CLT1, a putative GSH transporter located at the plastid envelope, while phytochelatins (PCs) are synthesized by phytochelatin synthetases (PCS) in the cytosol. Inorganic arsenic and cadmium form complexes with GSH and PCs. The GS_3_-As and GS_2_-Cd complexes might be sequestrated into the vacuole by unknown ABC transporters; PC-As can be delivered to the vacuole by AtABCC1, AtABCC2, and OsABCC1; and PC-Cd can be translocated into the vacuole by SpHMT1, AtABCC1, AtABCC2, and AtABCC3. Loss-of-function mutants for Arabidopsis *PCS1* and rice *CLT1* exhibited hypersensitivity to As and increased As translocation from roots to shoots due to the limited availability of a cytosolic As chelator in roots (Yang et al., 2016; Hayashi et al., 2017). Rice *abcc1* and *pcs1* mutants accumulated high levels of As in the grain due to the defect in vacuolar As sequestration in node I (Song et al., 2014a; Hayashi et al., 2017).

Vacuolar sequestration mediated by various transporters is important for reducing toxic metal(loid)s in the edible parts of plants. In rice, high levels of As were detected in vacuoles of root pericycle cells and companion cells of the nodal phloem, showing a strong co-localization with sulfur (Moore et al., 2011, 2014). A tonoplast-localized ATP-binding cassette (ABC) transporter, OsABCC1, was responsible for trapping As in the vacuoles by sequestering As-phytochelatin (PC) complex (Figure 1A, 2; Song et al., 2014b). Knockout of *OsABCC1* highly accumulated As in their grains due to a defect in the vacuolar compartmentalization of As in the phloem of the nodal vasculature, a critical tissue for distributing As to the grain (Song et al., 2014b).

PC is one kind of thiol-containing chelators synthesized from glutathione (GSH) by PC synthase (PCS) (Cobbett and Goldsbrough, 2002). Loss-of-function of the CRT-like transporter 1 (CLT1), a transporter that exports GSH from plastids to the cytosol, led to As hypersensitivity and increased root-to-shoot As translocation (Figure 2; Yang et al., 2016). A functionally defective mutant of *OsPCS1* exhibited increased As translocation to the grains from the node, indicating that this glutathione synthesis enzyme has a key role in As-PC sequestration in rice (Figure 2; Hayashi et al., 2017). Other transporters belonging to the C-type ABC superfamily, including budding yeast (*Saccharomyces cerevisiae*) cadmium factor 1 (ScYCF1), fission yeast (*Schizosaccharomyces pombe*) heavy metal tolerance 1 (SpHMT1), AtABCC1, AtABCC2, and AtABCC3 are crucial for vacuolar sequestration of As and/or Cd conjugated with GSH and/or PC (Ortiz et al., 1992; Ghosh et al., 1999; Song et al., 2010; Park et al., 2012; Brunetti et al., 2015).

Rice accessions harboring *OsHMA3a*, an *OsHMA3* allele from *indica* cultivars that results in loss of vacuolar Cd transport ability, showed much higher Cd translocation from roots to shoots and grains than did those with a functional *OsHMA3n* allele from the japonica cultivar Nipponbare (Ueno et al., 2010, 2011a; Miyadate et al., 2011). Overexpression of *OsHMA3n* dramatically decreased Cd concentration in rice shoots and grains (Ueno et al., 2010). This function was shown to be conserved in the closest homologs of OsHMA3 in Arabidopsis, soybean (*Glycine max*), and some Cd-hyperaccumulating species such as *Noccaea caerulescens* and *Sedum plumbizincicola* (Morel et al., 2009; Ueno et al., 2011b; Chao et al., 2012; Wang et al., 2012; Zhang et al., 2016; Liu et al., 2017). Other proteins belonging to the Cation Exchanger (CAX) and Metal Transporter Protein (MTP) families have also been implicated in Cd sequestration in plants (Martinoia et al., 2012; Shitan and Yazaki, 2013; Sharma et al., 2016), but it remains unclear whether they are involved in reducing Cd accumulation in the grain.

## Engineering Safer Cereals by Elevating Vacuolar Sequestration Capacity in Specific Tissues

Approaches to reduce As and Cd in cereals have been described in Arabidopsis, rice, and other plants. It was implicated that (i) reducing the uptake of toxic elements into the root, (ii) inhibiting the translocation of toxic elements from the root to the shoot, and (iii) preventing the distribution of toxic elements from to the grains are three critical steps that could decrease the levels of toxic metal(loid)s in grains (Zhao et al., 2010; Uraguchi and Fujiwara, 2013; Clemens and Ma, 2016; Awasthi et al., 2017; Chen et al., 2017; Lindsay and Maathuis, 2017).

Recently, two studies suggested that the tissue-specific expression of vacuolar transporters that sequester toxic metal(loid)s could strongly reduce Cd and As levels in the grain without reducing growth or yield (Deng et al., 2018; Shao et al., 2018). Transgenic rice plants were developed that expressed two different vacuolar As sequestration genes, *ScYCF1* and *OsABCC1*, under the control of the *RCc3* promoter in the root cortical and internode phloem cells, along with a gene for bacterial γ-glutamylcysteine synthetase gene, a key enzyme for glutathione synthesis, driven by the constitutive promoter. This tissue-specific expression of the two transporters was critical for reducing As concentrations in rice grains, as the level of As in the grains of plants ubiquitously expressing these genes was comparable to that of the WT (Figure 1A; Deng et al., 2018). The cortical cell-specific expression of *OsABCC1* and *ScYCF1* greatly reduced the translocation of As to shoots, due to increased vacuolar sequestration of As in root cortical cells, which constitute most of the rice root volume (Deng et al., 2018). This vacuolar sequestration of As within root cortical cells inhibits the radial translocation of As from the cortex to the endodermis, where OsLsi2, a major As efflux transporter localized to the proximal side of the endodermal layer, loads As into the xylem (Ma et al., 2008). More than 97% of the total As(III) taken up by the overexpression plant was trapped in the roots, compared to 87.5% in the wild type. At the reproductive growth stage, the triple overexpression plant showed an increased accumulation of As in internodes, and reduced As allocation to the upper leaves and grains (Deng et al., 2018). Furthermore, the As content was decreased by up to 70% in the grain of the overexpressors compared to the control (Deng et al., 2018). Despite this remarkable reduction, neither the growth nor the grain yield of the transgenic lines was impaired in a field test (Deng et al., 2018).

Rice lines in which a functional *OsHMA3* allele (*OsHMA3n*) was overexpressed under the control of a constitutive promoter, maize ubiquitin 1 (pZmUBI1), exhibited extremely low levels of Cd in the grain. Cd translocation from roots to shoots and grains was strongly inhibited in these transgenic plants (Ueno et al., 2010). Furthermore, their growth was similar to that of the control when grown under standard hydroponic conditions (Sasaki et al., 2014). Recently, Shao et al. (2018) developed another low Cd-accumulating transgenic rice line by expressing *OsHMA3n* under the control of the *OsHMA2* promoter (pOsHMA2) (Figure 1B). This resulted in the strong expression of *OsHMA3n* in root pericycle cells and resulted in a 60% reduction in the Cd root-to-shoot translocation rate. At the reproductive stage, Cd concentrations in the brown rice of transgenic lines were decreased to less than 10% of the control, indicating efficient compartmentalization of Cd into nodal vacuoles before it could be translocated to the grain (Shao et al., 2018). Neither the concentration of other minerals in the grain nor the grain yield was impaired in these lines (Shao et al., 2018).

Together, these results suggest that tissue-specific vacuolar sequestration of As and Cd is a useful strategy for developing crops that contain low levels of toxic metal(loid)s in the grain.

## Concluding Remarks and Future Perspectives

The recent progress highlighted here provides practical strategies for generating high-yield cereal crops with reduced concentrations of toxic metal(loid)s in their grains. Several studies indicate that enhancing vacuolar sequestration capacity is the most promising strategy to do so. This approach could be further improved by combining it with strategies that either improve release of As and Cd into the soil or inhibit their translocation to the shoot, as demonstrated by (Sun et al., 2018). However, our knowledge of how toxic metal(loid)s are translocated from the soil to grains is incomplete, even in rice. Furthermore, the molecular mechanisms underlying the accumulation and allocation of toxic minerals into grains in other cereal crops, including wheat, maize and barley, have yet to be identified. For instance, biochemical investigations using barley vacuoles demonstrated that ABC-type transporters function in Cd-PC and As-PC compartmentalization (Song et al., 2014a), however, the specific proteins involved have yet to be identified.

In addition to OsHMA3, OsABCC1, and their orthologs, at least two other groups of proteins are involved in detoxifying Cd and As. The first group is composed of proteins that regulate transport activity. For example, phosphorylation of Ser846 of AtABCC1 is required for As sequestration activity (Zhang et al., 2017) and mutation of the phosphorylatable Asp within the conserved DKTGT motif of AtHMA3 and AtHMA4 led to a severe inhibition of their Cd transport capabilities (Gravot et al., 2004; Verret et al., 2005). This suggests that, along with AtABCC1, OsHMA3 can be regulated by protein kinases, because the conserved DKTGT motif is also present in OsHMA3 (Ueno et al., 2010). It would be interesting to identify the interaction partners of OsABCC1 and OsHMA3, as they could be essential for reducing As and Cd accumulation in the grain. The second group consists of tonoplast-localized transporters and channels. For example, aquaporins are well-known channels that are permeable to As. Members of the nodulin-26-like intrinsic protein and plasma membrane-intrinsic protein subfamilies are critical for As(III) uptake and tolerance in Arabidopsis and rice (Lindsay and Maathuis, 2017). However, the physiological roles of tonoplast-intrinsic aquaporins in As sequestration have not been investigated in cereals. There have also not been functional studies to evaluate the importance of CAXs or MTPs for Cd sequestration in rice, wheat, or barley.

Although genetically modified (GM) rice plants with enhanced vacuolar sequestration capacity prevent toxic metal(loid) accumulation in grains, these lines may not be accepted by consumers who prefer non-GM food and may also be hindered by their legal classification (Dannenberg, 2009; Andersen et al., 2015; Østerberg et al., 2017). Natural allelic variants with elevated vacuolar sequestration capacity may facilitate the development of safer crops. For instance, a rare mutation in OsHMA4 leading to enhanced copper (Cu) transport activity associated with lower grain Cu in rice natural populations has been reported (Huang et al., 2016). Therefore, it would be worth identifying natural allelic variants in genes such as *OsHMA3*, *OsABCC1*, *OsPCS1*, and their novel regulation partners that result in enhanced vacuolar sequestration activity and synthesis of metal(loid)s chelators, using the rice and *Oryza* genome databases (Huang et al., 2012; Zhao et al., 2014; Ohyanagi et al., 2016; Sun et al., 2017; Wang et al., 2018). Unfortunately, no *OsHMA3* alleles with stronger Cd sequestration activity than that of OsHMA3n has been identified to date. Ueno et al. demonstrated that the highly conserved Arg80 of OsHMA3n is critical for its Cd transport activity. An His mutation at Arg80 in OsHMA3 may cause topological changes that lead to higher accumulation of Cd in the shoot and grain of some *Indica* rice accessions (Ueno et al., 2010, 2011a). In *Japonica* rice cultivars, an Arg substitution at Ser380 resulted in the loss-of-function of OsHMA3, which was associated with increased levels of Cd in the shoot and grain (Yan et al., 2016).

Although the function of *OsHMA3* homologs in other cereals has not been identified, HvHMA3 has been implicated in Cd accumulation in barley grains by genome-wide association mapping (Wu et al., 2015). Genetic markers can be designed based on the sequences of functional *HMA3* alleles and employed for marker-assisted selection to generate low Cd-accumulating cereal cultivars. Once identified, natural rice accessions with a higher vacuolar sequestration capacity can be easily used as breeding donors to develop crops that do not accumulate toxic metal(loid)s. Additionally, genotypes exhibiting altered kinase activities that modulate the activity of the critical transporters may have further reductions in toxic metal(loid)s content in the grain. These strategies will become increasingly viable as the genomic information of other cereals becomes available.

## Author Contributions

FD and W-YS conceptualized the review. FD and W-YS wrote the review. FD, MY, EM, and W-YS did final editing of the manuscript.

## Conflict of Interest Statement

The authors declare that the research was conducted in the absence of any commercial or financial relationships that could be construed as a potential conflict of interest.
